# Associations between body composition measurements of obesity and radiographic osteoarthritis in older adults: Data from the Dong-gu Study

**DOI:** 10.1186/s12891-016-1040-9

**Published:** 2016-04-29

**Authors:** Lihui Wen, Ji-Hyoun Kang, Yi-Rang Yim, Ji-Eun Kim, Jeong-Won Lee, Kyung-Eun Lee, Dong-Jin Park, Tae-Jong Kim, Yong-Wook Park, Sun-Seog Kweon, Young-Hoon Lee, Yong-Woon Yun, Min-Ho Shin, Shin-Seok Lee

**Affiliations:** Department of Rheumatology, Chonnam National University Medical School & Hospital, 42 Jebong-ro, Dong-gu, Gwangju 501-757 Republic of Korea; Department of Biomedical Sciences, Chonnam National University Medical School, Gwangju, Republic of Korea; Department of Preventive Medicine, Chonnam National University Medical School, 160 Baekseo-ro, Dong-gu, Gwangju 501-746 Republic of Korea; Jeonnam Regional Cancer Center, Chonnam National University Hwasun Hospital, Hwasun, Republic of Korea; Department of Preventive Medicine & Institute of Wonkwang Medical Science, Wonkwang University College of Medicine, Iksan, Republic of Korea; Gwangju-Jeonnam Regional Cardiocerebrovascular Center, Chonnam National University Hospital, Gwangju, Republic of Korea

**Keywords:** Osteoarthritis, Obesity, Radiography

## Abstract

**Background:**

We examined the effects of fat deposition on radiographic osteoarthritis (OA) to determine the role of obesity in the pathogenesis of radiographic OA.

**Methods:**

Data were taken from the Dong-gu cohort, a cross-sectional study of 2,367 subjects. Baseline characteristics, waist circumference (WC), waist-to-hip ratio (WHR), fat mass, and fat percentage were collected, along with X-rays of the knees and hands. Total knee and hand radiographic OA scores were summed using a semi-quantitative grading system, and then stratified by gender using a multiple linear regression model.

**Results:**

After adjusting for confounders, weight was the only factor significantly associated with knee radiographic OA, regardless of gender (all *p* < 0.01). Regarding the hand, fat percentage had the largest effect on radiographic OA in males (*p* = 0.008), while WHR was the most significant factor in females (*p* = 0.001). For the knee, fat mass was the most important factor for radiographic OA in males (*p* = 0.001), while in females, body mass index was the most important factor (*p* < 0.001). Among the variables, only fat percentage was significantly related to both hand and knee radiographic OA in both genders (all *p* < 0.01).

**Conclusions:**

Regardless of gender, weight was significantly associated with knee radiographic OA. Otherwise, fat deposition correlated with hand and knee radiographic OA in both genders, while the distribution of fat tissue was significantly associated with hand and knee radiographic OA only in females.

## Background

Osteoarthritis (OA) is a heterogeneous group of diseases with differing pathogenesis in different joints [1]. The onset of OA is uncommon before the age of 40, but the prevalence rises rapidly with age thereafter [2]. With increasing life expectancy, nearly one-third of middle-aged adults will have OA by the year 2030 [3]. Among all the known risk factors, including mechanical, biochemical, and genetic factors, obesity is one of the main risk factors for the incidence and prevalence of OA [4]. However, the mechanisms linking obesity and OA still remain unclear.

One reason for this lack of clarity may be that obesity is typically defined by a body mass index (BMI) [5], which measures only height and weight, with no consideration of other obesity-related characteristics, such as the distribution of fat [waist circumference (WC), waist-to-hip ratio (WHR)] or fat deposition (fat mass, fat percentage), and does not discriminate adipose from non-adipose mass. In contrast, fat deposition and distribution may serve as better indicators of the metabolic activity of adipose tissue, and can be used as indirect measures of inflammatory cytokine secretion. Greater insight into the relationships between these body composition measurements and OA will be useful for investigation of the mechanisms by which obesity and OA interact.

Another possible reason for the lack of a clear mechanism linking fat deposition and OA is the variability in fat metabolism, which differs according to anatomical location and gender. Fat deposition occurs primarily in the upper body in males, whereas fat is more likely to accumulate in the lower body in females. These differences in adipose deposition exert significant physiological effects, which may affect the development of OA [6]. Previous studies assessing the role of body composition in OA focused only on knee OA, and did not consider gender, resulting in inconclusive findings [7–11]. However, other studies which examined non-weight-bearing joints, such as the hand, revealed no association with fat percentage or WC, and provided conflicting results in terms of WHR [12–14]. Recently, Visser et al. [15] found that knee OA was associated with weight and fat-free mass, whereas hand OA was associated with metabolic syndrome. However, the diagnosis of hand and knee OA in that study was based on clinical criteria alone, with no X-rays available, and the subjects were not separated by gender.

To gain further insight into the role of obesity in the pathology of radiographic OA, it is important to investigate the effects of fat deposition on radiographic OA. In our cross-sectional, population-based study, we evaluated the relationships between obesity-related characteristics and radiographic OA of the hand and knee in an Asian population. We hypothesized that the body composition measurements would be variously associated with hand and knee radiographic OA in males and females.

## Methods

### Subjects

The Dong-gu Study is an ongoing prospective study to investigate the prevalence, incidence, and risk factors for chronic diseases in 9,260 subjects, aged 50 years or older, as previously described in detail [16]. Baseline data were collected during the period 2007 to 2010 in Dong-gu, Gwangju Metropolitan City, Republic of Korea. Hand and knee X-rays were obtained from only 2,489 subjects, from a total of 2,516 participants in the 2009 baseline examination. Of those, 51 subjects with a past history of knee replacement surgery or knee amputation, and 71 subjects with missing data on lifestyle factors, were excluded. Finally, the remaining 2,367 subjects were included in the analysis. All participants provided informed consent at the time of enrolment into the study, and the study was approved the Institutional Review Board of Chonnam National University Hospital (IRB No. CNUH-2015-041).

### Covariates

The smoking status, alcohol consumption, and educational background of study participants were assessed using a standardized questionnaire. Smoking status was classified as current smokers (smoked ≥ 100 cigarettes in their lifetime and currently a smoker) and non-current smokers (smoked < 100 cigarettes in their lifetime and not currently smoking). Alcohol consumption (within the past 12 months) was classified as current drinkers and non-current drinkers. Education was categorized into middle school or less and high school or more. Diabetes mellitus was defined as a fasting blood glucose > 126 mg/dL or the use of hypoglycemic medication. Hypertension was defined as a systolic blood pressure ≥ 140 mmHg or a diastolic blood pressure ≥ 90 mmHg, or the use of antihypertensive medication.

### Body composition measurements

Height was measured to the nearest 0.1 cm, and weight was recorded to the nearest 0.1 kg. BMI was defined as weight (in kilograms) divided by height (in meters squared). Body composition (fat mass and fat percentage) was measured by bioelectrical impedance analysis using a calibrated InBody 520 body composition analyzer (Biospace Co. Ltd., Seoul, Korea). WC was measured with the subject standing, at the level midway between the lower rib margin and the iliac crest. Hip circumference was measured at the fullest point around the buttocks. WHR was defined as the ratio of waist to hip circumferences.

### Assessment of radiographic features

X-rays of both knees and hands were obtained using a computed radiography X-ray system. When measuring the knees, anteroposterior extended-view weight-bearing radiographs were obtained. When measuring the hands, all participants pronated the hands with the palmar surface in contact with the cassette and the fingers slightly spread. All images were anteroposterior radiographs, and either knees or hands were evaluated on a single X-ray film. Radiographs were scored by two trained observers blinded to clinical details, using a semi-quantitative grading system, and with reference to the Atlas of Standard Radiographs of Arthritis [17]. The initial scores of the two observers were compared and the radiographs were then reviewed by a third independent observer in the case of any disagreement. The interobserver and intraobserver reliability were tested on a subgroup of the Dong-gu cohort study. The evaluation was performed twice on 100 randomly-chosen radiographs (50 radiographs of the knee, 50 radiographs of the hand) by the same observers 1 month apart. Kappa statistics were used to assess interobserver (*k* = 0.79 – 0.89) and intraobserver (*k* = 0.85 – 0.92) reproducibility.

Individual radiographic features were recorded for the hand (distal interphalangeal joint [DIP], proximal interphalangeal joint [PIP], trapeziometacarpal joint [CMC], interphalangeal joint of the thumb [IP], and naviculotrapezial joint [NTJ]), and knee (medial compartment, lateral compartment, tibial component, and femoral component). The extent of primary osteophytes and joint space narrowing (JSN) at primary sites (DIP, PIP, CMC, IP, NTJ, medial and lateral compartments for knee JSN, and medial femoral condyle, medial tibial plateau, lateral femoral condyle, and lateral tibial plateau for knee osteophytes) were graded from 0 to 3 (i.e., 0 = normal, 1 = mild change, 2 = moderate change, 3 = severe change). Other abnormalities (malalignment, erosion, subchondral sclerosis, subchondral cysts, and attrition) and OA at other sites (IP and NTJ) were graded as absent (0) or present (1). Using this semi-quantitative grading system, total OA scores were computed by summing the scores of individual radiographic features.

The semi-quantitative grading system yielded total OA scores (max. 42) for the knee. The components thereof, including osteophyte (max. 24), JSN (max. 12), tibial attrition (max. 4), and sclerosis (max. 2) scores, were calculated and listed as means ± SDs. For the hand, osteoarthritic total score (max. 70), and osteophyte (max. 22), JSN (max. 22), subchondral cyst (max. 4), sclerosis (max. 6), erosion (max. 10), and malalignment (max. 6) scores were calculated. Of these, the osteophyte and JSN scores comprised the major proportions of the total scores for the knee (85.7 %) and hand (62.9 %).

### Statistical analysis

Student’s *t*-tests or *χ*^2^ tests were used to compare means or proportions of baseline data, divided by gender. Linear regression analyses were performed with or without adjustment for confounding factors to evaluate the associations between body composition measurements and radiographic OA scores in hands and knees. Model 1 was unadjusted. Model 2 was adjusted for age, current smoking, current alcohol consumption, and education. Model 3 was further adjusted for hypertension and diabetes mellitus. Partial eta-squared (%) and standard beta coefficients were estimated to assess the effect size of each body composition measurement. A *p* value of < 0.05 (two-tailed) or a 95 % confidence interval, not including the null point, was regarded as statistically significant. All statistical analyses were performed with the SPSS for Windows software (ver. 20.0; SPSS Inc., Chicago, IL, USA).

## Results

### Baseline characteristics of enrolled subjects

Table 1 presents the baseline characteristics of the study participants. In total, 2,367 subjects were enrolled in the study, with a mean age of 64.0 ± 8.2 years. Of the total subjects, 1,340 (56.6 %) were female, and their mean age (63.3 ± 8.2 years) was significantly lower than that of the male subjects (64.9 ± 8.1 years; *p* < 0.001). The body composition measurements according to gender showed significant differences; the mean BMI, mean fat mass, and mean fat percentage of the female group were all higher than those of the male group (*p* < 0.001), whereas the mean weight, mean WC, and mean WHR of the female group were lower than those of the male group (*p* < 0.001). Other social demographic characteristics (current smoking, current alcohol consumption, education, hypertension, and diabetes mellitus) were also found to be significantly lower in the female group (*p* < 0.001).

**Table 1:** Baseline characteristics of the subjects stratified by gender

	Total	Male	Female	*P* value
N	2367	1027	1340	
Age, years	64.0 ± 8.2	64.9 ± 8.1	63.3 ± 8.2	<0.001
Weight, kg	61.5 ± 9.4	66.1 ± 9.0	58.0 ± 8.1	<0.001
BMI, kg/m^2^	24.4 ± 2.9	24.1 ± 2.8	24.7 ± 3.0	<0.001
Current smoking (%)	291 (12.3)	267 (26.0)	24 (1.8)	<0.001
Current alcohol consumption (%)	1186 (50.1)	700 (68.2)	486 (36.3)	<0.001
Education (high school or more) (%)	762 (32.2)	491 (47.8)	271 (20.2)	<0.001
Hypertension (%)	1153 (48.7)	519 (50.5)	634 (47.3)	<0.001
Diabetes mellitus (%)	483 (20.4)	244 (23.8)	239 (17.8)	<0.001
Waist circumference, cm	86.0 ± 8.1	86.8 ± 7.4	85.4 ± 8.5	<0.001
Waist-to-hip ratio	0.92 ± 0.06	0.93 ± 0.05	0.91 ± 0.06	<0.001
Fat mass, kg	19.3 ± 5.8	17.3 ± 5.4	20.8 ± 5.6	<0.001
Fat percentage (%)	31.2 ± 7.5	25.7 ± 5.8	35.4 ± 5.8	<0.001

Abbreviations: *BMI* body mass index

### Associations between body composition measurements and hand radiographic OA

Table 2 presents the associations between body composition measurements and hand radiographic OA in both genders. Using simple linear regression analysis with no adjustment, we found that weight was associated negatively with hand radiographic OA. However, in analyses adjusted for confounders, weight was not associated with hand radiographic OA in both genders. Outside of the effects of weight on OA, fat mass and fat percentage were significantly associated with hand radiographic OA in males after adjustment, and BMI, WC, WHR, and fat percentage was significantly associated with hand radiographic OA in females in unadjusted and adjusted analyses. Among these factors, fat percentage in males was found to have the largest impact on hand radiographic OA (standard beta = 0.076, eta = 0.007), whereas WHR had the largest effect on hand radiographic OA in females (standard beta = 0.077, eta = 0.009).

**Table 2:** Relationships between body composition measurements and hand osteoarthritis

		Model 1				Model 2				Model 3			
		Beta	St. Beta	Eta	*P* value	Beta	St. Beta	Eta	*P* value	Beta	St. Beta	Eta	*P* value
Male	Weight, kg	–0.083	–0.133	0.018	<0.001	0.002	0.003	0.000	0.916	–0.001	–0.001	0.000	0.976
	BMI, kg/m^2^	–0.108	–0.053	0.003	0.087	0.064	0.031	0.001	0.258	0.054	0.026	0.001	0.360
	Waist circumference, cm	0.019	0.025	0.001	0.427	0.035	0.046	0.003	0.091	0.034	0.045	0.002	0.115
	Waist-to-hip ratio	4.642	0.040	0.002	0.202	0.087	0.001	0.000	0.978	–0.319	–0.003	0.000	0.922
	Fat mass, kg	0.056	0.054	0.003	0.082	0.062	0.060	0.005	0.029	0.059	0.057	0.004	0.047
	Fat percentage (%)	0.147	0.152	0.023	<0.001	0.077	0.079	0.008	0.004	0.074	0.076	0.007	0.008
Female	Weight, kg	–0.077	–0.093	0.009	0.001	0.015	0.018	0.000	0.421	0.012	0.014	0.000	0.521
	BMI, kg/m^2^	0.140	0.062	0.004	0.023	0.116	0.052	0.004	0.017	0.117	0.052	0.004	0.020
	Waist circumference, cm	0.088	0.112	0.013	<0.001	0.052	0.066	0.007	0.002	0.055	0.069	0.007	0.002
	Waist-to-hip ratio	13.574	0.127	0.016	<0.001	7.748	0.073	0.008	0.001	8.240	0.077	0.009	0.001
	Fat mass, kg	0.058	0.049	0.002	0.075	0.050	0.042	0.003	0.049	0.049	0.042	0.003	0.059
	Fat percentage (%)	0.184	0.161	0.026	<0.001	0.069	0.061	0.006	0.005	0.071	0.062	0.006	0.005

Abbreviations: *BMI* body mass index, *St. Beta* standard beta coefficients, *Eta* partial eta-squared

Model 1, no adjustment

Model 2, adjusted by age, current smoking, current alcohol consumption, education

Model 3, adjusted by age, current smoking, current alcohol consumption, education, hypertension, and diabetes mellitus

### Associations between body composition measurements and knee radiographic OA

The associations between body composition measurements and knee radiographic OA were analyzed in the same manner as described above (Table 3). In simple linear regression analysis with no adjustment, only fat mass and fat percentage were associated significantly with knee radiographic OA in males. In an analysis adjusted for all confounding factors, the relationship between weight, BMI, WC, fat mass, and fat percentage, and knee radiographic OA in males was statistically significant. In contrast, the body composition measurements were all found to be significantly related to knee radiographic OA in females, regardless of adjustment by confounders. Fat mass had strongest effect on knee radiographic OA in males (standard beta = 0.105, eta = 0.012), whereas BMI was the most significant factor in females (standard beta = 0.218, eta = 0.059).

**Table 3:** Relationships between body composition measurements and knee osteoarthritis

		Model 1				Model 2				Model 3			
		Beta	St. Beta	Eta	*P* value	Beta	St. Beta	Eta	*P* value	Beta	St. Beta	Eta	*P* value
Male	Weight, kg	–0.028	–0.043	0.002	0.165	0.037	0.057	0.003	0.064	0.045	0.070	0.005	0.027
	BMI, kg/m^2^	0.045	0.022	0.000	0.488	0.173	0.082	0.007	0.006	0.193	0.092	0.009	0.003
	Waist circumference, cm	0.036	0.046	0.002	0.141	0.049	0.063	0.004	0.032	0.059	0.075	0.006	0.014
	Waist-to-hip ratio	2.635	0.022	0.000	0.484	–0.764	–0.006	0.000	0.829	–0.068	–0.001	0.000	0.985
	Fat mass, kg	0.094	0.088	0.008	0.005	0.098	0.092	0.009	0.002	0.113	0.105	0.012	0.001
	Fat percentage (%)	0.143	0.142	0.020	<0.001	0.092	0.092	0.009	0.002	0.102	0.102	0.011	0.001
Female	Weight, kg	0.065	0.070	0.005	0.011	0.154	0.165	0.035	<0.001	0.163	0.175	0.037	<0.001
	BMI, kg/m^2^	0.539	0.212	0.045	<0.001	0.522	0.206	0.055	<0.001	0.555	0.218	0.059	<0.001
	Waist circumference, cm	0.187	0.210	0.044	<0.001	0.155	0.175	0.040	<0.001	0.167	0.187	0.044	<0.001
	Waist-to-hip ratio	21.068	0.174	0.030	<0.001	15.635	0.130	0.022	<0.001	16.815	0.139	0.024	<0.001
	Fat mass, kg	0.249	0.186	0.035	<0.001	0.242	0.181	0.043	<0.001	0.256	0.191	0.046	<0.001
	Fat percentage (%)	0.314	0.242	0.059	<0.001	0.211	0.163	0.034	<0.001	0.220	0.170	0.036	<0.001

Abbreviations: *BMI* body mass index, *St. Beta* standard beta coefficients, *Eta* partial eta–squared

Model 1, no adjustment

Model 2, adjusted by age, current smoking, current alcohol consumption, education

Model 3, adjusted by age, current smoking, current alcohol consumption, education, hypertension, and diabetes mellitus

## Discussion

In this population of healthy community-based older adults, weight was significantly associated with radiographic OA only in the knee joint, regardless of gender. Otherwise, fat deposition correlated with hand and knee radiographic OA in both genders, while the distribution of fat tissue was significantly related to hand and knee radiographic OA in females only.

As expected, our results confirmed previous findings of a positive relationship between weight and knee OA [7, 11, 15, 18], supporting the concept of excessive mechanical stress on the joint surface of obese individuals, resulting in damaged joint tissue. Moreover, it can be inferred that the compression of cartilage might activate mechanoreceptors on chondrocytes, inducing signaling cascades and leading to the synthesis of inflammatory mediators and tissue remodeling [4, 19]. On the other hand, weight did not affect radiographic OA of the hand, which is a non-weight-bearing joint. This observation confirmed that the mechanical factors of obesity appear to affect only weight-bearing joints.

In our study, hand radiographic OA in females, and knee radiographic OA in both genders, showed positive relationships with BMI. Whereas, in males, BMI had no effect on hand radiographic OA. In a recent systematic review, Yusuf et al. also found that BMI tended to be positively associated with hand OA in most studies [20], although no subgroup analysis according to gender was performed. In this case, a third of the included studies that concerned only women revealed a consistent positive association between BMI and hand OA, while other mixed-gender studies showed inconsistent associations. These results suggest that BMI is associated positively with radiographic hand OA outcomes in females only. As BMI represents a distinct measurement independent of weight, which was not associated with hand OA in either gender, these findings suggest a biochemical effect of obesity, which may be involved in the generation of hand OA.

In this study, we examined the distribution of fat tissue and found that both WC and WHR were associated with hand and knee radiographic OA in females, in line with other recent studies [14, 21–25]. One reason for this may be that visceral fat has been suggested to secrete bioactive cytokines, which play a role in the pathogenesis of OA [26]. Another hypothesis is that increased WC may have biomechanical effects that could alter gait and stance, affecting the quadriceps angle and varus or valgus deformities [27]. In our study, these results appeared to be restricted to the female subjects. According to the population-based KORA Survey 2000 [28], WC and WHR are strongly associated with low-grade systemic inflammation, especially in women, indicating the effect of gender differences on the risk of OA. Similar analyses conducted as part of the Genetics of OA and Lifestyle (GOAL) study [24] also revealed clear gender differences, with the risk of lower-limb OA associated strongly with WC and WHR in women. However, WC and WHR were not associated with an increased risk of OA, regardless of gender. The results of a subsequent Korean cohort study further supported these findings, with WC related significantly to knee OA only in female subjects [23]. Finally, in a population-based prospective cohort study [21], the risk of knee OA was associated more strongly with increases in WC and WHR in female patients. As WHR is associated negatively with female sex hormones [29], the changes seen in postmenopausal women may represent a significant risk factor for OA in this population. Furthermore, higher C-reactive protein (CRP) concentrations seen in women as a result of higher body fat percentages are also likely to affect OA risk, relative to men [30]. Thus, the distribution of fat tissue tends to correlate more with radiographic OA in females.

To our knowledge, this study is the first to report positive associations between fat deposition and radiographic OA in both the hand and knee joints. In general, fat mass is considered to affect knee OA through two mechanisms: 1) weight-bearing effects (the exertion of more mechanical stress on the knee joints) and 2) weight-independent effects (e.g., secretion of inflammatory cytokines, which negatively affect knee joints). However, as our analyses revealed no obvious distinction between OA in the hand (a non-weight-bearing joint) and OA in the knee (a weight-bearing joint) in relation to fat mass, weight-independent effects should be regarded as the primary mechanisms driving pathology in both hand and knee OA. With regarding to the weight independent effect, fat mass is known to be negatively associated with tibial cartilage volume and increased tibiofemoral cartilage defects [7]. In addition, the association of fat deposition with OA may be related to the increased adipose production of CRP or other inflammatory mediators, including interleukin-6, that have previously been shown to be associated with OA [31]. Unsurprisingly, reduced exercise due to a large amount of fat also can promote the prevalence and incidence of OA [32]. Meanwhile, reviewing the previous studies, our findings are also in line with those from Visser et al. where the fat mass and fat percentage were associated with hand OA [25], although hand OA were diagnosed using clinical criteria, no X-rays were available, and the subjects were not separated by gender. When knee OA was included in their investigation, fat mass and fat percentage were also found to be related to all types of OA [15]. Therefore, it can be summarized that fat deposition correlates with hand and knee OA in both genders; in our study, this correlation was more obvious in the male subjects.

The strengths of our study are as follows. First, we included more detailed body composition measurements to analyze the various relationships with radiographic OA, and we classified all subjects by gender, thus providing further insights into the effects of gender differences on radiographic OA. Second, we simultaneously compared radiographic OA between weight-bearing and non-weight-bearing joints, and employed a semi-quantitative grading system to evaluate the severity of radiographic OA. Third, we made adjustments for several confounding factors, such as current smoking, current alcohol consumption, education, hypertension, and diabetes mellitus, which are known risk factors for OA; many other studies did not collect or adjust for these data. Fourth, a unique contribution of this study is the consideration of these questions in an Asian population. This population is largely under-studied in OA research, and evidence suggests that body composition and fat distribution may confer differential risk in Asian populations as compared with white populations. Last, the Dong-gu Study has been conducted with a large number of community-dwelling older adults; the original subjects included more than 10,000 people, a representative sample of the general Korean population.

However, the present study also has some limitations. Firstly, the cross-sectional nature of this study limited our ability to directly assess the interaction between obesity and radiographic OA, such as whether mechanical stress caused by obesity increases damage to the weight-bearing joints, or whether pain, functional decline, and stiffness caused by OA add to the progression of obesity. As fat deposits in obese patients are major sources of pro- and anti-inflammatory adipokines, these tissues may also play an important role in OA pathogenesis [33–35]. Finally, previous studies have shown relationships between metabolic syndromes, such as diabetes mellitus and atherosclerosis, and OA, with obesity identified as a major underlying factor driving metabolic syndromes [36–39], suggesting a direct link between these important pathologies. Further studies are planned to directly assess each of these outcomes. Secondly, the semi-quantitative grading system is still not precise enough, similar to the K-L grading system used to distinguish the severity of radiographic features on visual inspection. Thirdly, more potential covariates including exercise were not collected in this cross-sectional study. Therefore, we were unable to provide direct insight into the role of these variables in this population. We believe that this study will promote future clinical studies to evaluate the relationship between obesity and radiographic OA.

## Conclusion

Our results suggest that obesity has an adverse effect on radiographic OA; weight, in the form of mechanical stress, was found to be associated with knee radiographic OA in both genders. Fat deposition had a greater effect on hand and knee radiographic OA, regardless of gender. Fat distribution, however, was important for hand and knee radiographic OA in females only.

### Ethics and consent to participate

All participants provided informed consent at the time of enrolment into the study, and the study was approved the Institutional Review Board of Chonnam National University Hospital (IRB No. CNUH-2015-041).

### Consent to publish

Not applicable.

### Availability of data and materials

All supporting data can be provided upon request to the authors.

## Abbreviations

BMI: body mass index
CMC: trapeziometacarpal
DIP: distal interphalangeal
IP: interphalangeal
JSN: joint space narrowing
NTJ: naviculotrapezial
OA: osteoarthritis
PIP: proximal interphalangeal
WC: waist circumference
WHR: waist-to-hip ratio
